# Methylthio-Aspochalasins from a Marine-Derived Fungus *Aspergillus* sp.

**DOI:** 10.3390/md12105124

**Published:** 2014-09-30

**Authors:** Ying Liu, Shizhe Zhao, Wanjing Ding, Pinmei Wang, Xianwen Yang, Jinzhong Xu

**Affiliations:** 1 Institute of Marine Biology, Ocean College, Zhejiang University, Hangzhou 310058, China; E-Mails: liuy620@163.com (Y.L.); zhao.shizhe@163.com (S.Z.); dwj1988555@126.com (W.D.); wangpinmei@zju.edu.cn (P.W.); 2 CAS Key Laboratory of Tropical Marine Bio-resources and Ecology, South China Sea Institute of Oceanology, Chinese Academy of Sciences, Guangzhou 510301, China; E-Mail: yangxw76@163.com

**Keywords:** gut fungi, *Aspergillus* sp., aspochalasin, methylthiol, cytotoxicity

## Abstract

Two novel aspochalasins, 20-β-methylthio-aspochalsin Q (named as aspochalasin V), (**1**) and aspochalasin W (**2**), were isolated from culture broth of *Aspergillus* sp., which was found in the gut of a marine isopod *Ligia oceanica*. The structures were determined on the basis of NMR and mass spectral data analysis. This is the first report about methylthio-substituted aspochalasin derivatives. Cytotoxicity against the prostate cancer PC3 cell line and HCT116 cell line was assayed using the MTT method. Apochalasin V showed moderate activity at IC_50_ values of 30.4 and 39.2 μM, respectively.

## 1. Introduction

Fungi are important producers of marine natural products and most of them were found from algae and sponge [1]. Actually various microorganisms including fungi were found in the gut of marine isopods [2,3,4,5,6]. These gut symbioses showed some ecological roles such as chemical defense [7]. Therefore gut microbes including fungi may be an interesting source of bioactive marine natural products. In fact, an actinomycete strain IFB-A01, capable of producing new neuraminidase inhibitors, was isolated from the gut of the shrimp *Penasus orientalis* [8]. In our investigation into new bioactive metabolites of marine gut fungi, one strain (Z-4) was isolated from the gut of the marine isopod *Ligia oceanica*. In its culture broth two aspochalasins containing unusual methylthiol groups were found. Aspochalasins are a subgroup of cytochalasans, consisting of a macrocylic ring, isoindolone moiety and a 2-methyl-propyl side chain. So far, more than 20 compounds, including aspochalasins A–U and Z [9,10,11,12,13,14], have been reported. These compounds contained almost the same carbon skeleton and their structural diversity included a double bond shift (C5/C6 or C6/C7) in the isoindolone unit together with ketone, hydroxyl, methoxyl and double bond reduction in the macrocylic ring. Aspochalasins showed various bioactivities such as antibiotic [15,16,17], cytotoxicity [13,18], anti-HIV [19], TNF-alpha [20] and melanogenesis [21] inhibitors. Herein, we describe the isolation, structural elucidation, and cytotoxic activity of these two new aspochalasins.

## 2. Results and Discussion

**Figure 1:**
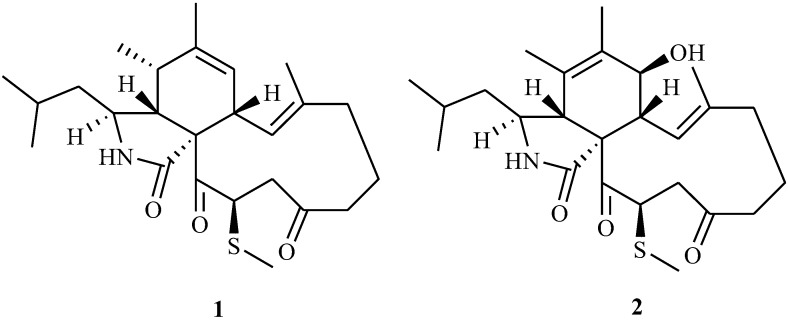
Chemical structures of compounds **1** and **2**.

Compound **1** was isolated as a colorless solid. The ESI-HRMS showed a quasi-molecular ion peak at *m*/*z* 432.2572 for [M + H]^+^, indicating the molecular formula of compound **1** is C_25_H_37_NO_3_S (calcd. 432.2528 for C_25_H_38_NO_3_S) by combination with 1D NMR data. IR spectrum showed obvious peaks at 3350, 1687 and 1700 cm^−1^. ^13^C NMR data together with DEPT 135 and DEPT 90 spectra revealed that **1** contained twenty-five carbons, including two ketone carbonyl signals (δ_C_ 208.1 and 200.5), one amide carbonyl signal (δ_C_ 177.2), four olefinic carbons (δ_C_ 141.8, 138.9, 126.5 and 125.2), five aliphatic methylene carbons (δ_C_ 49.9, 44.4, 41.9, 40.2 and 19.8), six aliphatic methine carbons (δ_C_ 54.1, 52.7, 46.2, 46.0, 37.2 and 25.7), six methyl signals (δ_C_ 24.2, 21.9, 20.1, 15.1, 14.0 and 10.8) together with one quaternary carbon (δ_C_ 66.1). These data indicated that **1** was an aspochalasin derivative [15], which contained two pairs of double bonds and two ketones. Comparison of ^1^H NMR data, especially olefinic proton signals (δ_H_ 6.29 (d, *J* = 10.8 Hz) and 5.30 (brs)) and methyl proton signals (δ_H_ 1.80 (s), 1.36 (s), 1.28 (d, *J* = 7.2 Hz), 0.94 (d, *J* = 6.8 Hz) and 0.92 (d, *J* = 6.8 Hz)) with those of reported aspochalasin derivatives, indicated that **1** was similar to aspochalasin Q ^11^ expect for one up-field methyl signal (δ_H_ 1.85, δ_C_ 10.8). Only one correlation from δ_H_ 1.85 to δ_C_ 46.0 in the HMBC spectrum means this methyl connected with backbone through S atom. ^1^H-^1^H COSY correlations among δ_H_ 5.03 (H-20), δ_H_ 3.00 (H-19) and δ_H_ 2.65 (H-19) together with HMBC correlation from δ_H_ 3.00 to δ_C_ 40.2 (C-17) revealed that this methylthiol group connected with C-20 of aspochalasin Q (Figure 1). Proton and carbon signals were fully attributed by 2D NMR data analysis (^1^H-^1^H COSY, HMQC and HMBC) (Table 1).

**Table 1:** NMR data for compounds **1** (methanol-*d*_4_) and **2** (CDCl_3_).

NO.	1		2	
	^13^C	^1^H (mult., *J* in Hz)	^13^C	^1^H (mult., *J* in Hz)
1	177.2		174.7	
3	52.7	3.17 (m)	56.2	3.25 (m)
4	54.1	2.55 (brs)	50.3	3.21 (s)
5	37.2	2.55 (brs)	127.0	
6	141.8		131.6	
7	126.5	5.30 (brs)	69.4	3.88 (d, 9.6)
8	46.2	3.00 (m)	49.1	2.36 (t,10.4)
9	66.1		59.6	
10	49.9	1.21,( m), 1.47 ( m)	45.5	1.22,( m), 1.47 ( m)
11	14.0	1.28 (d, 6.8)	17.7	1.74 (s)
12	20.1	1.80 (s)	13.9	1.74 (s)
13	125.2	6.29 (d, 10.8)	121.4	6.22 (d,10.8)
14	138.9		144.7	
15	41.9	2.19 (brd, 8.0), 1.96 (td,12.0, 4.0)	41.3	2.19 (m), 2.24 (m)
16	19.8	2.29 (dd, 18.0,8.0 ), 1.56 (m)	19.2	2.32 (m), 2.24 (m)
17	40.2	2.70 (dd, 10.4, 18.6), 2.26 (m)	40.0	2.69 (dd,10.4, 18.0), 2.17 (m)
18	208.1		205.1	
19	44.4	2.65 (dd,11.6,2.4), 3.00 (m)	44.1	3.08 (t,11.6), 2.57 (dd,11.6,2.0m)
20	46.0	5.03 (dd,12.8,2.4)	44.2	4.77 (dd,12.4,2.0)
21	200.5		196.2	
22	25.7	1.69 (m)	24.8	1.62 (m)
23	21.9	0.94 (d,6.8)	21.5	0.90 (d,6.8)
24	24.2	0.92 (d,6.8)	23.3	0.89 (d,6.8)
25	15.1	1.36 (brs)	15.6	1.45 (brs)
26	10.8	1.85 (s)	10.9	1.84 (s)

**Figure 2:**
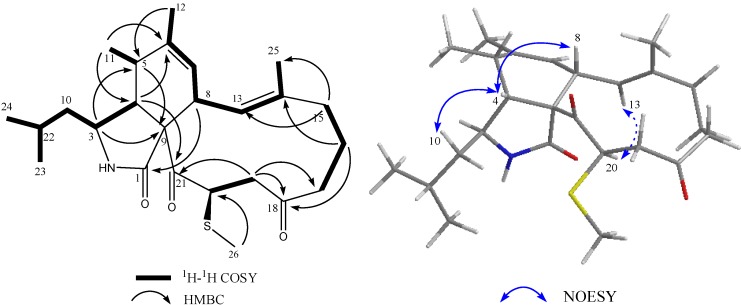
^1^H-^1^H COSY, key HMBC and selected NOESY correlations for **1**.

So far, in all natural cytochalasans, the cyclohexane/isoindole ring junction and the macrocyclic ring have been described to have *cis*- and *trans*-stereochemistry, respectively [18]. It is reported that this is the absolute configuration of cytochalasans because of the diastereofacial selectivity of the cyclo-addition reaction during the biosynthesis, which assigned the absolute configurations for C-3, C-4, C-8 and C-9 as 3*S*, 4*R*, 8*R*, and 9*R*, respectively. NOESY correlations between proton signals of δ_H_ 2.55 (H-4) and δ_H_ 1.21 (H-10β), δ_H_ 2.55 (H-4) and δ_H_ 3.00 (H-8) in **1** supported this result. Otherwise NOESY spectrum of **1** showed obvious correlation from the proton at δ_H_ 5.03 (H-20) to that at δ_H_ 6.29 (H-13), by which H-20 was determined in an α-oriention through the model structure of MM2 minimized energy calculation of Chemdraw 3D software (Figure 2). Thus compound **1** was identified as 20-β-methylthio-aspochalasin Q, named as aspochalasin V (Figure 1).

**Figure 3:**
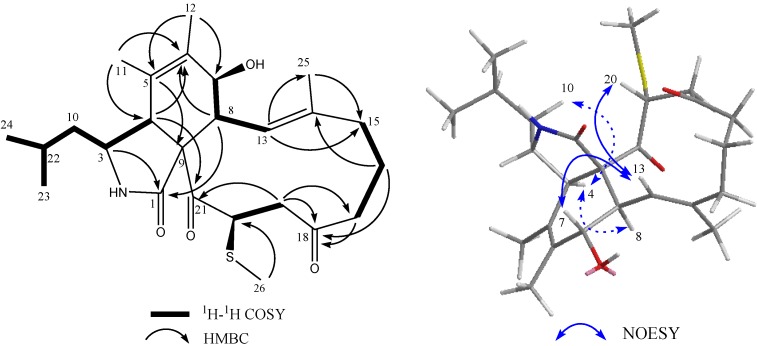
^1^H-^1^H COSY, key HMBC and selected NOESY correlations for **2**.

Compound **2** was obtained as a colorless solid. The ESI-HRMS showed a quasi-molecular ion peaks at *m*/*z* 448.2522 and *m*/*z* 917.4780 for [M + H]^+^ and [2M + Na]^+^, respectively, indicating the molecular formula of compound **2** is C_25_H_37_NO_4_S (calcd. 448.2516 for C_25_H_38_NO_4_S) by combination with 1D NMR data. Similar to those of **1**, 1D NMR data of **2** also indicated a methylthio-substituted aspochalasin and **2** contained one oxygen atom more than **1**. Different from **1**, the presence of one oxygenated aliphatic methine (δ_H_ 3.88, d, *J* = 9.6 Hz, δ_C_ 69.4), one olefinic quaternary carbon (δ_C_ 131.6) and one singlet methyl proton signals (δ_H_ 1.74, s, δ_C_ 17.7) together with the absences of one olefinic methine signal (δ_H_ 5.30, δ_C_ 126.5 in **1**), one aliphatic methine (δ_H_ 2.55, δ_C_ 37.2 in **1**) and one doublet methyl (δ_H_ 1.28, δ_C_ 14.0 in **1**) indicated that one double bond at C-6/C-7 in **1** moved to C-5/C-6 and C-7 was oxygenated in **2**. Similar backbone was reported in aspochalasin L^19^. Relative ^1^H-^1^H COSY and HMBC correlations (Figure 3) revealed that the methylthiol group was also connected with C-20 in compound **2**. One the basis of unambiguous absolute configurations for C-3, C-4, C-8 and C-9 in aspochalasins, the stereochemistry of C-7 was determined as an *R*-configuration (β-OH) due to the coupling constant (9.6 Hz) between H-7/H-8 and NOESY correlation between H-7/H-13. The orientation of methylthiol group was determined as β through the obvious NOESY cross peak between H-20/H-13 in **2** (Figure 3). Thus absolute structure of compound **2** was determined and named as aspochalasin W (Figure 1). Proton and carbon signals were fully attributed by 2D NMR data analysis (^1^H-^1^H COSY, HMQC and HMBC) (Table 1).

Cytochalasans are a major group of fungal metabolites, nearly 100 of which were reported previously. To our best knowledge, this is the first example of methylthio-substituted cytochalasans. Methythio-substitution usually occurs in diketopiperazine derivatives of fungi and is derived from reductive methylation of disulfide linkage [22]. As for the biosynthesis of the methylthiol group in **1** and **2**, methanethiol etherification reaction is a possible pathway because of existence of methioninase in *Aspergillus sp* [23].

Compound **1** was tested for cytotoxicity against prostate cancer cell line PC3 and the HCT116 cell line through the MTT method and showed moderate growth inhibitory activities at IC_50_ values of 30.4 and 39.2 μM, respectively. Compound **2** was not subjected to other spectral tests and bioassay because the compound was lost after the NMR and MS data collection. In comparison with reported data [11,18,19], the methylthiol group showed no effect for cytotoxicity of aspochalasins.

## 3. Experimental

### 3.1. General Experimental Procedures

NMR spectra were recorded in CDCl_3_ (ALDRICH, St. Louis, MO, USA) with tetramethylsilane as an internal standard or Methanol-*d_4_* (ALDRICH, St. Louis, MO, USA), using a Bruker DPX 400 MHz NMR spectrometer (Brucker, Ettlingen, Germany). HR-ESIMS data was recorded on an Agilent 6224 TOF LC/MS. Infrared (IR) spectroscopy was performed on a Bruker V-22 spectrometer (Bruker Corporation, Fällanden, Switzerland). UV spectrum was recorded on a HITACHI U-3010 spectrometer (HITACHI, Tokyo, Japan). Optical rotation was measured on a JASCO P-1010 digital polarimeter (JASCO, Tokyo, Japan).

### 3.2. Fungus Material and Compound Isolation

Marine isopod *Ligia oceanica* was collected in seaside of Dinghai in Zhoushan, Zhejiang Province of China in December 2011. The gut was isolated and cut into pieces and cultured in 2216E agar media (QingDao Hopebio-Technology Co., Ltd, Qingdao, China) dissolved in artificial seawater. A fungal colony (Z-4) was isolated and determined as *Aspergillus* sp*.* by 18S rDNA analysis (see Supplementary Information). The fungus was preserved in China Center for Type Culture Collection (CCTCC No. M2013631). The strain (Z-4) was cultured in one hundred 500-mL Erlenmeyer-flasks containing 200 mL of 2216E liquid media (QingDao Hopebio-Technology Co., Ltd., Qingdao, China) for 4 weeks at room temperature. The culture broth was filtered and extracted with EtOAc to obtain 10 g fungal secondary metabolites extract. Extract was subjected to Silica gel column chromatography eluted in gradient by CH_2_Cl_2_–MeOH (100:1–0:100) and separated into 9 fractions. Fraction 4 (CH_2_Cl_2_–MeOH 9:1) was separated by silica gel column with cyclohexane–EtOAc gradient elution to obtain 9 subfractions. Subfractions (Z4-8) and (Z4-7) were purified by semi-preparative ODS-HPLC (COSMOSIL PACKED COLUMN, 5C18-MS-II column, 10ID × 250 mm, Nacalai Tesque, Kyoto, Japan) to obtain compounds **1** (30 mg, 58% MeCN-H_2_O) and **2** (2 mg, 48% MeCN-H_2_O), respectively.

20-β-methylthio-aspochalsin Q (**1**): white powder, [α]^27^_D_ +169.6 (*c* 5.0, MeOH); UV (MeOH) λ_max_ (log ε): 204 (3.30), 290 (2.15) nm. IR ν_max_ 3350, 1687, 1700 cm^−1^. ^1^H and ^13^C NMR: see Table 1 and Supplementary Information. ESI-HRMS *m*/*z* 432.2572 [M + H]^+^ (calcd. 432.2528 for C_25_H_38_NO_3_S).

Aspochalasin W (**2**): white powder, ^1^H and ^13^C NMR: see Table 1 and Supplementary Information. ESI-HRMS *m*/*z* 448.2522 [M + H]^+^, *m*/*z* 917.4780 [2M + Na]^+^, (calcd. 448.2516 for C_25_H_38_NO_4_S).

### 3.3. Cytotoxicity against Cancer Cell Lines

The cytotoxicity was measured by 3-(4,5-dimethylthiazol-2-yl)-2,5-diphenyltetrazolium bromide (MTT) assay. Tumor cell lines were seeded in 96-well plates (4 × 10^3^ per well in 100 μL). After 24 h of incubation in the appropriate medium, cells were treated with different concentrations (100 μM, 50 μM, 25 μM, 12.5 μM, 6.25 μM, 3.125 μM) for another 72 h. Afterwards, MTT solution (5.0 mg/mL in RPMI-1640 Media, Sigma, St. Louis, MO, USA) was added (20 μL/well) and then plates were incubated for another 4 h at 37 °C. The purple formazan crystals were dissolved in 100 μL dimethyl sulfoxide (DMSO). After 5 min, the plates were read on a Multiskan Spectrum (Themo Instruments Inc., Waltham, MA, USA) at 570 nm. The IC_50_ values were obtained using the software of Dose–Effect Analysis with Microcomputers and were defined as concentration of drug causing 50% inhibition in absorbance compared with control cells. Assays were performed in triplicate in three independent experiments.

## 4. Conclusions

The chemical investigation of the culture broth of a marine gut fungus *Aspergillus* sp. led to the isolation of two novel aspochalasin compounds, which contained unusual methylthiol groups at the C-20 position. Their stereochemistry was determined through 2D-NMR data analysis and compared with reported aspochalasins. The result of bioassays indicted that the aspochalasin Compound **1** showed moderate cytotoxicity against PC3 and HCT116 cells.

## Supplementary Files

Supplementary File 1

## Abbreviations

ESI-HRMS: Electron Spray Ionization-High Resolution Mass Spectrum
DEPT: Distortionless Enhancement by Polarization Transfer
HMBC: Heteronuclear Multiple Bond Correlation
COSY: Correlation Spectroscopy
HMQC: Heteronuclear Multiple Quantum Correlation
NOESY: Nuclear Overhauser Enhancement Spectroscopy
MM2: Minimize Model
