# Supplementary material for: Methylthio-Aspochalasins from a Marine-Derived Fungus Aspergillus sp
Source: Mar Drugs. 2014 Sep 30;12(10):5124–31. doi: 10.3390/md12105124 (PMC4210888; doi:10.3390/md12105124)
Supplement: Supplementary File 1 [file marinedrugs-12-05124-s001.pdf]

Supplementary Information

1. 18S rDNA Sequence Information of the Strain Z-4

The amplification by the primers of NS1 (5′-GTAGTCATATGCTTGTCTC-3′) and NS8 (5′-TCCGCAGGTTCACCTACGGA-3′) obtained 18S rDNA sequence information of Z-4 as follows:

GGTCGCATGTCTAGTATATGCACTTTATACCGTGAAACTGCGAATGGCTCATTAAATCAGTTATCGTTTATT
TGATAGTACCTTACTACATGGATACCTGTGGTAATTCTAGAGCTAATACATGCTAAAAACCCCGACTTCGG
AAGGGGTGTATTTATTAGATAAAAAACCAATGCCCTTCGGGGCTCCTTGGTGATTCATAATAACTTAACGA
ATCGCATGGCCTTGCGCCGGCGATGGTTCATTCAAATTTCTGCCCTATCAACTTTCGATGGTAGGATAGTG
GCCTACCATGGTGGCAACGGGTAACGGGGAATTAGGGTTCGATTCCGGAGAGGGAGCCTGAGAAACGG
CTACCACATCCAAGGAAGGCAGCAGGCGCGCAAATTACCCAATCCCGACACGGGGAGGTAGTGACAATA
AATACTGATACGGGGCTCTTTTGGGTCTCGTAATTGGAATGAGTACAATTTAAATCCCTTAACGAGGAACA
ATTGGAGGGCAAGTCTGGTGCCAGCAGCCGCGGTAATTCCAGCTCCAATAGCGTATATTAAAGTTGTTGC
AGTTAAAAAGCTCGTAGTTGAACCTTGGGTCTGGCTGGCCGGTCCGCCTCACCGCGAGTACTGGTCCGG
CTGGACCTTTCCTTCTGGGGAATCCCATGGCCTTCACTGGCTGTGGGGGGAACCAGGACTTTTACTGTGA
AAAAATTAGAGTGTTCAAAGCAGGCCTTTGCTCGAATACATTAGCATGGAATAATAGAATAGGACGTGCG
GTTCTATTTTGTTGGTTTCTAGGACCGCCGTAATGATTAATAGGGATAGTCGGGGGCGTCAGTATTCAGCT
GTCAGAGGTGAAATTCTTGGATTTGCTGAAGACTAACTACTGCGAAAGCATTCGCCAAGGATGTTTTCAT
TAATCAGGGAACGAAAGTTAGGGGATCGAACGATCAGATACCGTCGTAGTCTTAACCATAAACTATGCCG
ACTAGGGATCGGGCGGTGTTTCTATGATGACCCGCTCGGCACCTTACGAGAAATCAAAGTTTTTGGGTTC
TGGGGGGAGTATGGTCGCAAGGCTGAAACTTAAAGAAATTGACGGAAGGGCACCACAAGGCGTGGAGC
CTGCGGCTTAATTTGACTCAACACGGGGAAACTCACCAGGTCCAGACAAAATAAGGATTGACAGATTGA
GAGCTCTTTCTTGATCTTTTGGATGGTGGTGCATGGCCGTTCTTAGTTGGTGGAGTGATTTGTCTGCTTAA
TTGCGATAACGAACGAGACCTCGGCCCTTAAATAGCCCGGTCCGCATTTGCGGGCCGCTGGCTTCTTAGG
GGGACTATCGGCTCAAGCCGATGGAAGTGCGCGGCAATAACAGGTCTGTGATGCCCTTAGATGTTCTGGG
CCGCACGCGCGCTACACTGACAGGGTCAGCGAGTACATCACCTTGGCCGAGAGGTCTGGGTAATCTTGT
TAAACCCTGTCGTGCTGGGGATAGAGCATTGCAATTATTGCTCTTCAACGAGGAATGCCTAGTAGGCACG
AGTCATCAGCTCGTGCCGATTACGTCCCTGCCCTTTGTACACACCGCCCGTCGCTACTACCGATTGAATGG
CTCGGTGAGGCCTTCGGACTGGCTCAGGAGGGTTGGCAACGACCCCCCAGAGCCGGAAAGTTGGTCAA
ACCCGGTCATAGAGAAGAAAGTATT

Figure S1. Comparing the Sequence with Those in NCBI Gene Bank.

Accession	Description	Max score	Total score	Query coverage	E value	Max ident
NW_001884680.1	Aspergillus oryzae RIB40 contig SC206	3051	3051	99%	0.0	99%
NW_001849579.1	Aspergillus oryzae RIB40 contig rDNA_te13	3051	3051	99%	0.0	99%
NT_166520.1	Aspergillus niger CBS 513.88 supercontig An03	3046	1.025e+04	99%	0.0	99%
NC_007197.1	Aspergillus fumigatus Af293 chromosome 4, whole genome shotgu	3046	4439	99%	0.0	99%
NT_165929.1	Aspergillus terreus NIH2624 scaffold_6 genomic scaffold, whole ge	2972	2972	99%	0.0	98%
NW_001510400.1	Aspergillus clavatus NRRL 1 1099423829788 genomic scaffold, wh	2918	2918	94%	0.0	99%
NW_002990118.1	Talaromyces stipitatus ATCC 10500 scf_1105507295515, whole g	2915	2915	99%	0.0	98%
NW_003101676.1	Ajellomyces dermatitidis SLH14081 genomic scaffold supercont1.7	2824	1.493e+04	99%	0.0	97%
NW_003217216.1	Paracoccidioides brasiliensis Pb01 supercont1.72 genomic scaffolc	2813	9289	99%	0.0	97%
NW_003217281.1	Paracoccidioides brasiliensis Pb01 supercont1.7 genomic scaffold,	2813	8440	99%	0.0	97%

2. NMR Spectra of Novel Compounds

Figure S2. Proton NMR for Compound **1**.

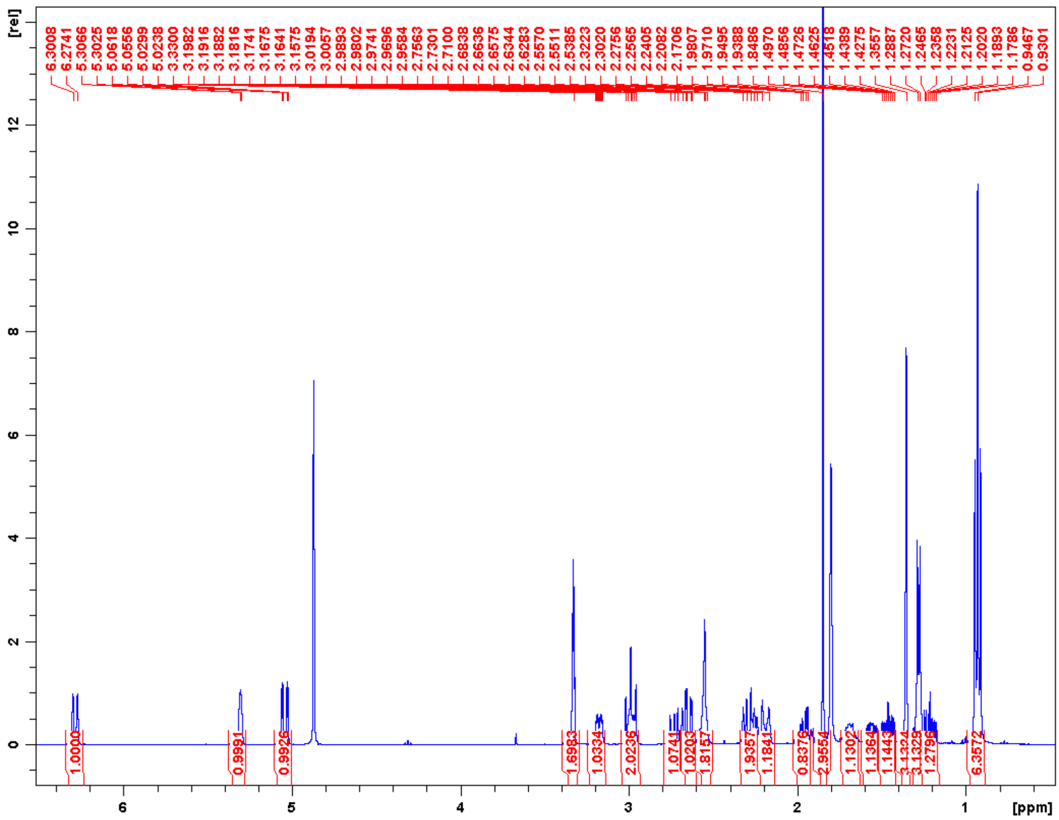

Figure S3. Carbon NMR for Compound **1**.

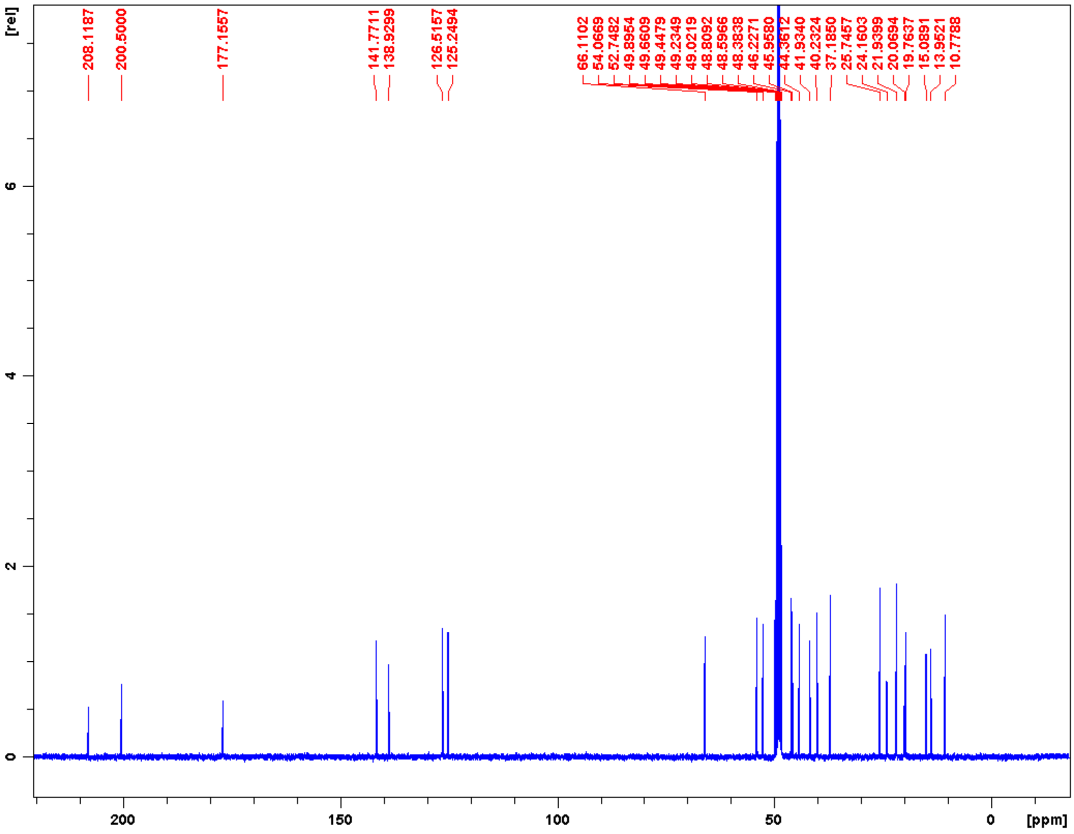

Figure S4. DEPT135 Spectrum for Compound **1**.

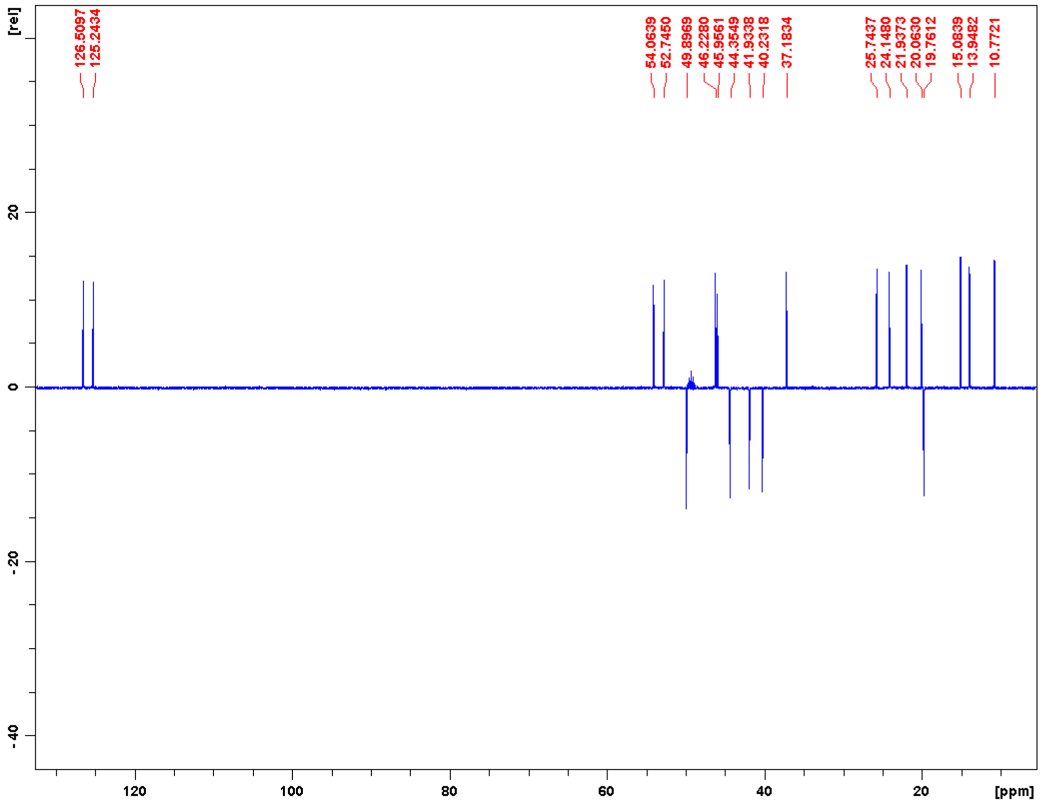

Figure S5. DEPT90 Spectrum for Compound **1**.

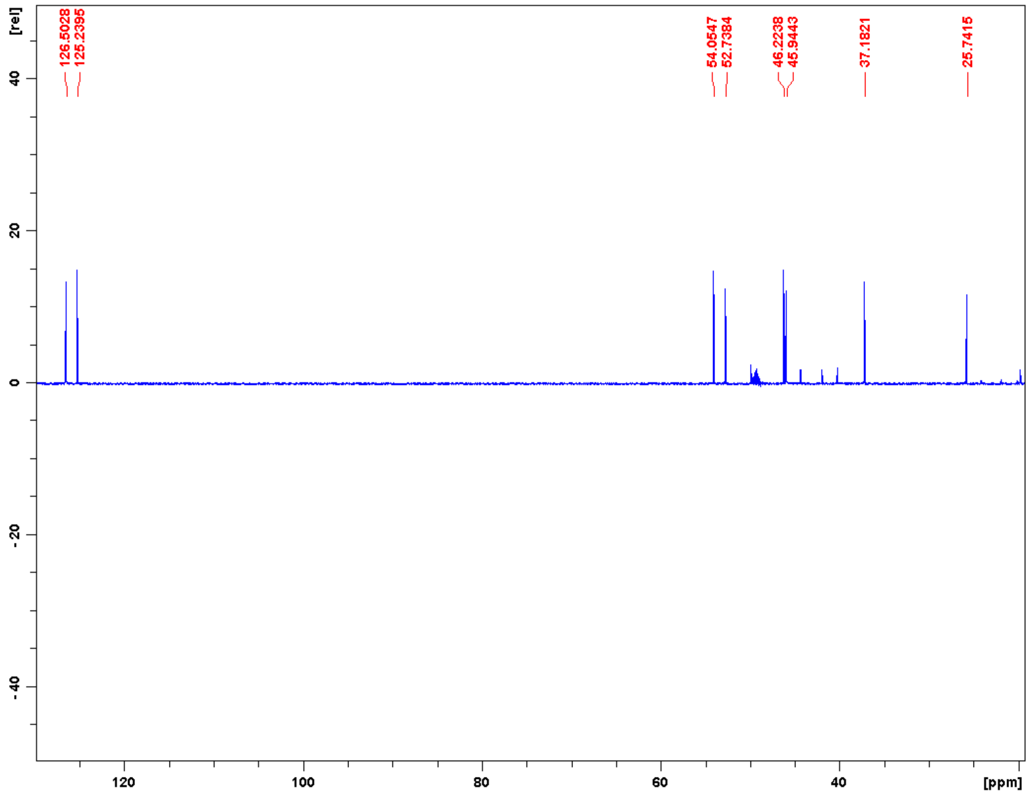

Figure S6. ^{1}H-^{1}H COSY for Compound **1**.

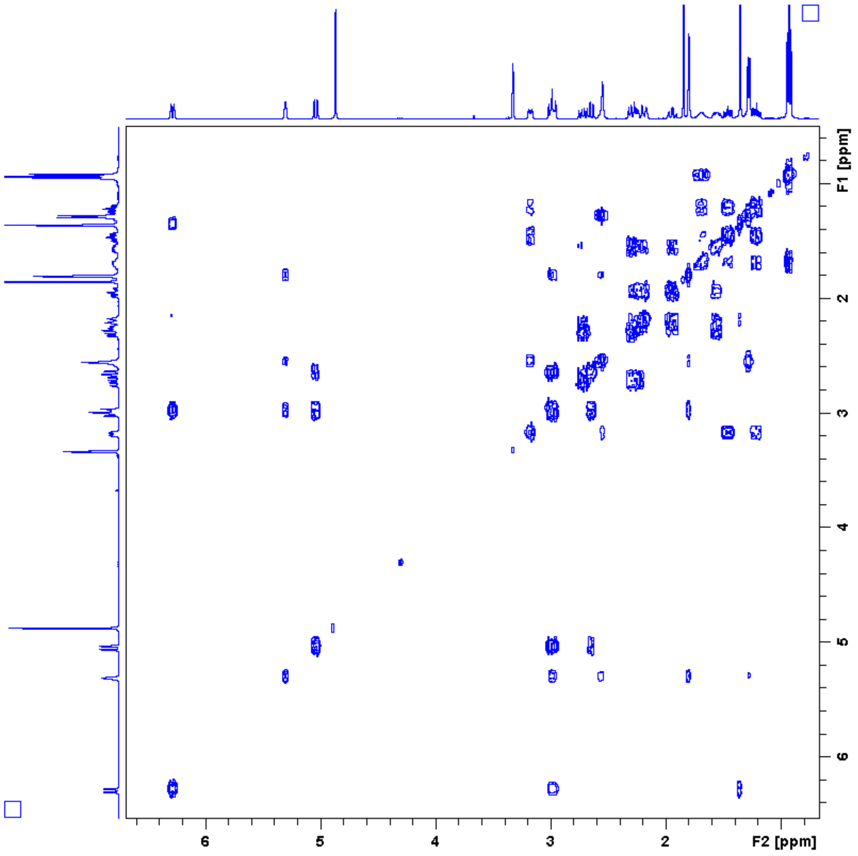

Figure S7. HMQC for Compound **1**.

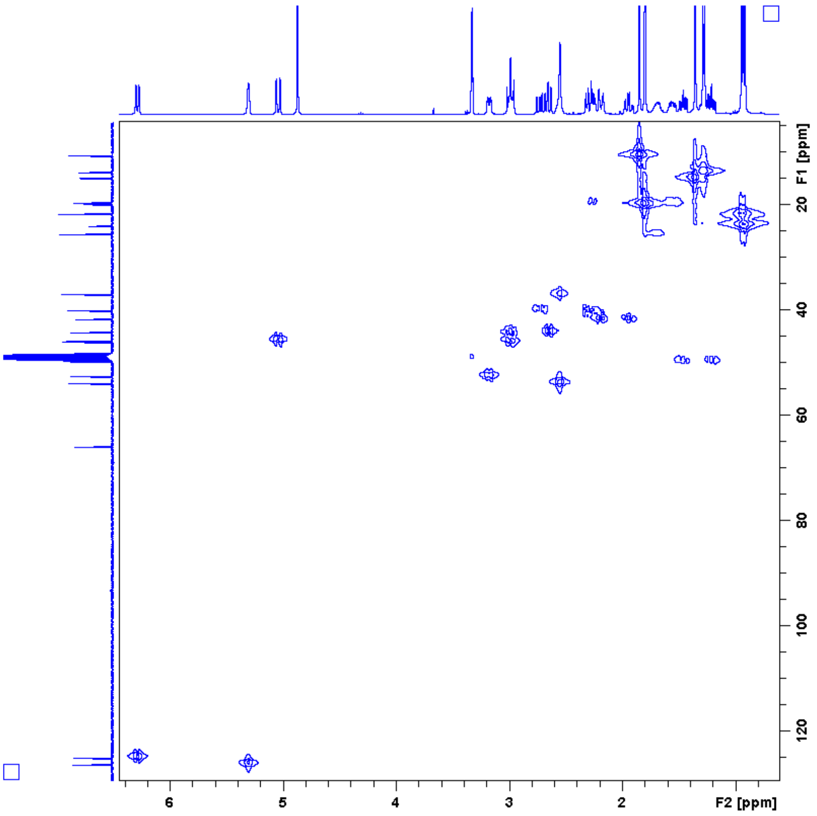

Figure S8. HMBC for Compound **1**.

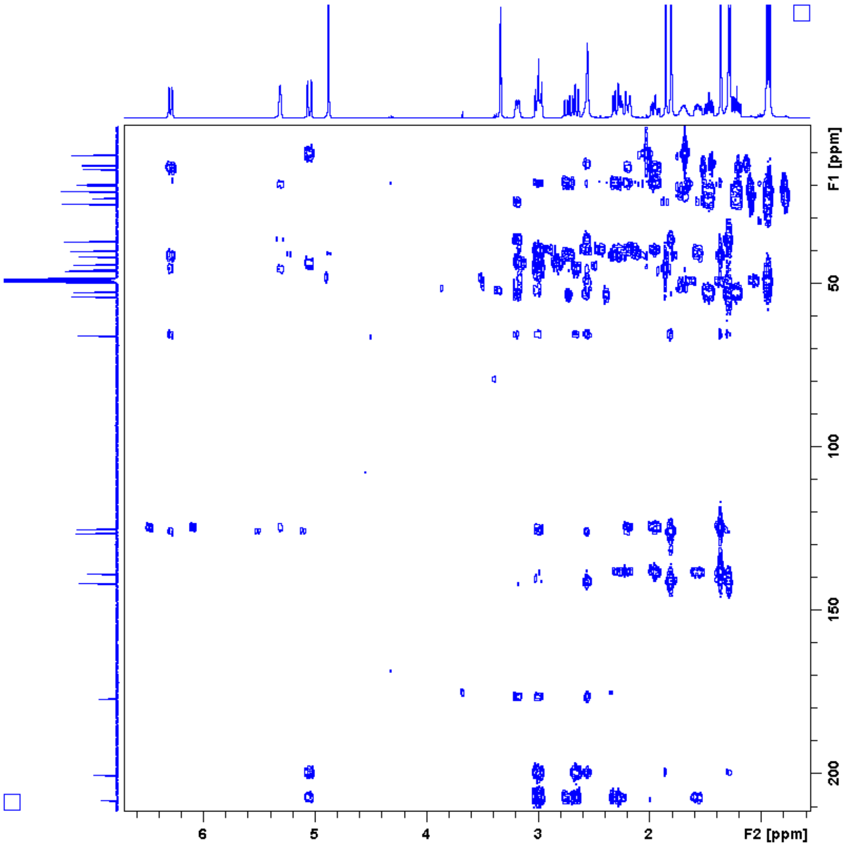

Figure S9. NOESY for Compound **1**.

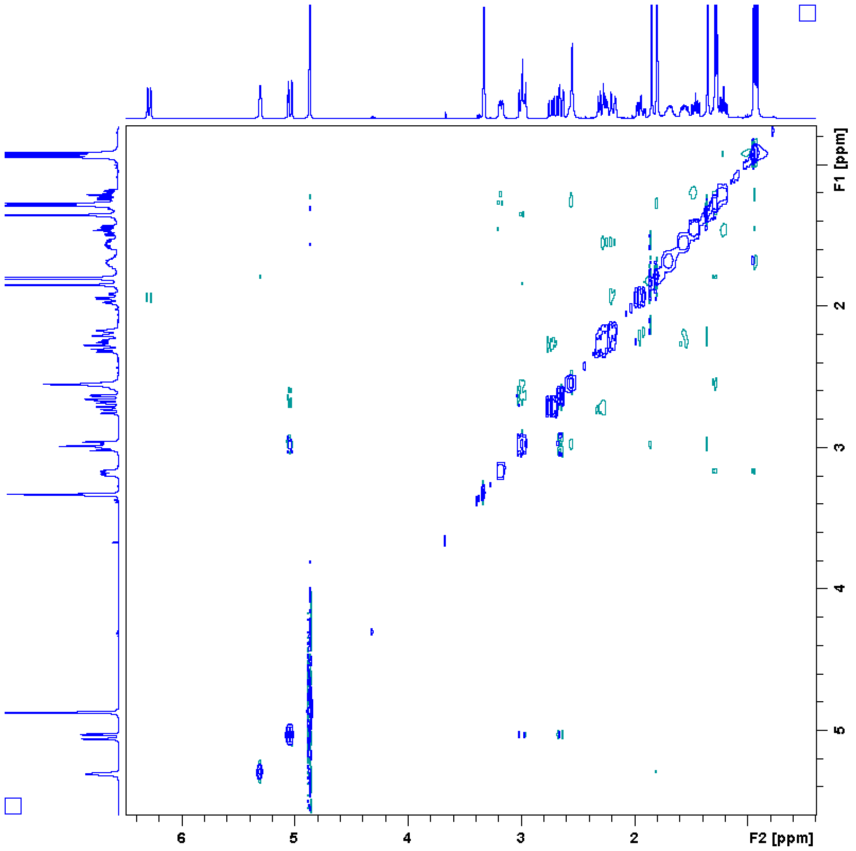

Figure S10. Proton NMR for Compound **2**.

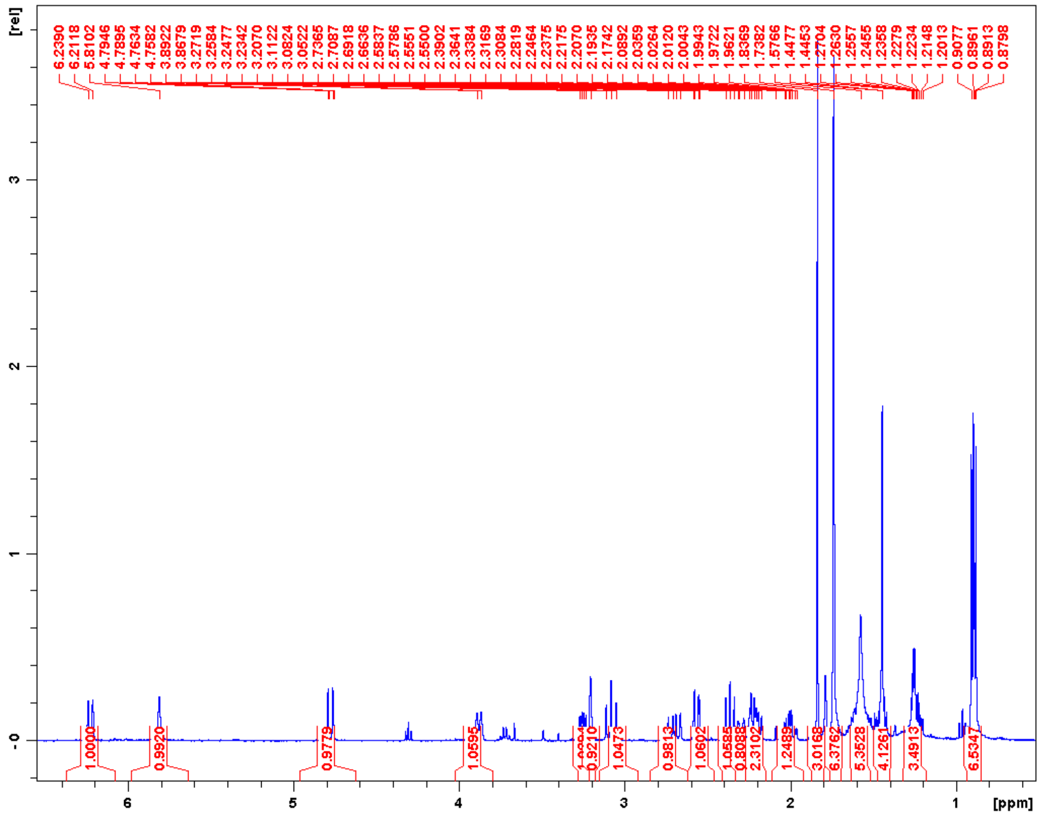

Figure S11. Carbon NMR for Compound **2**.

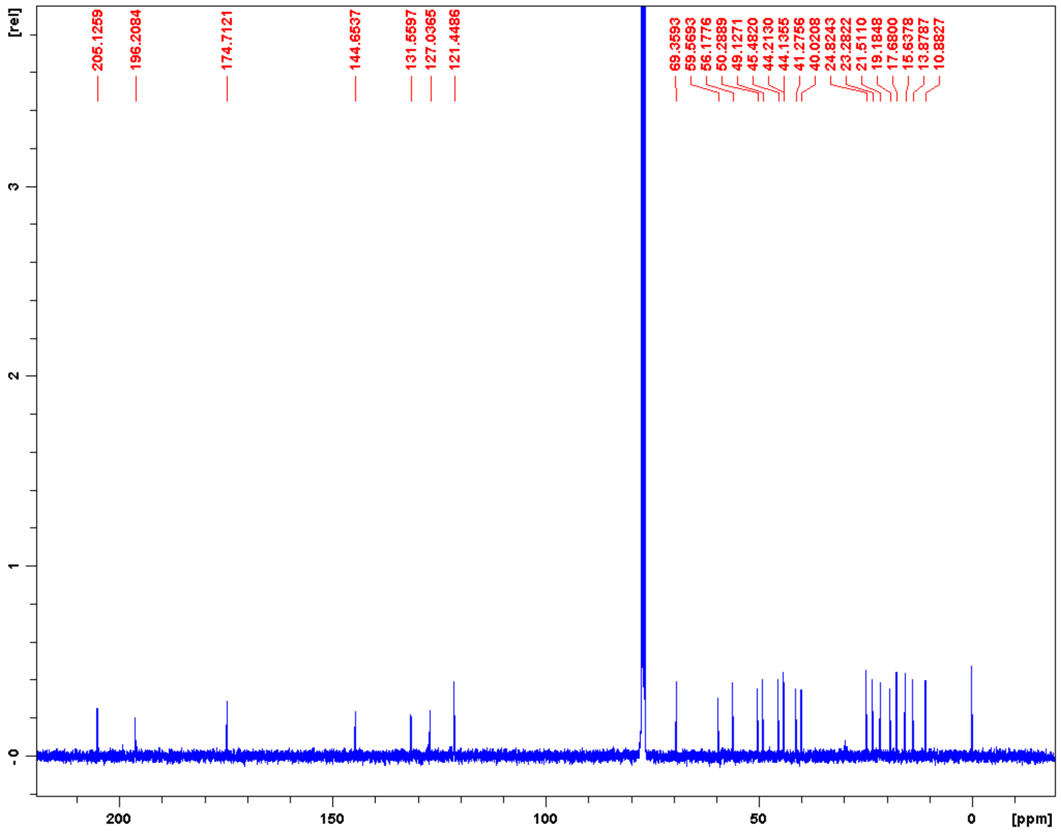

Figure S12. DEPT135 Spectrum for Compound **2**.

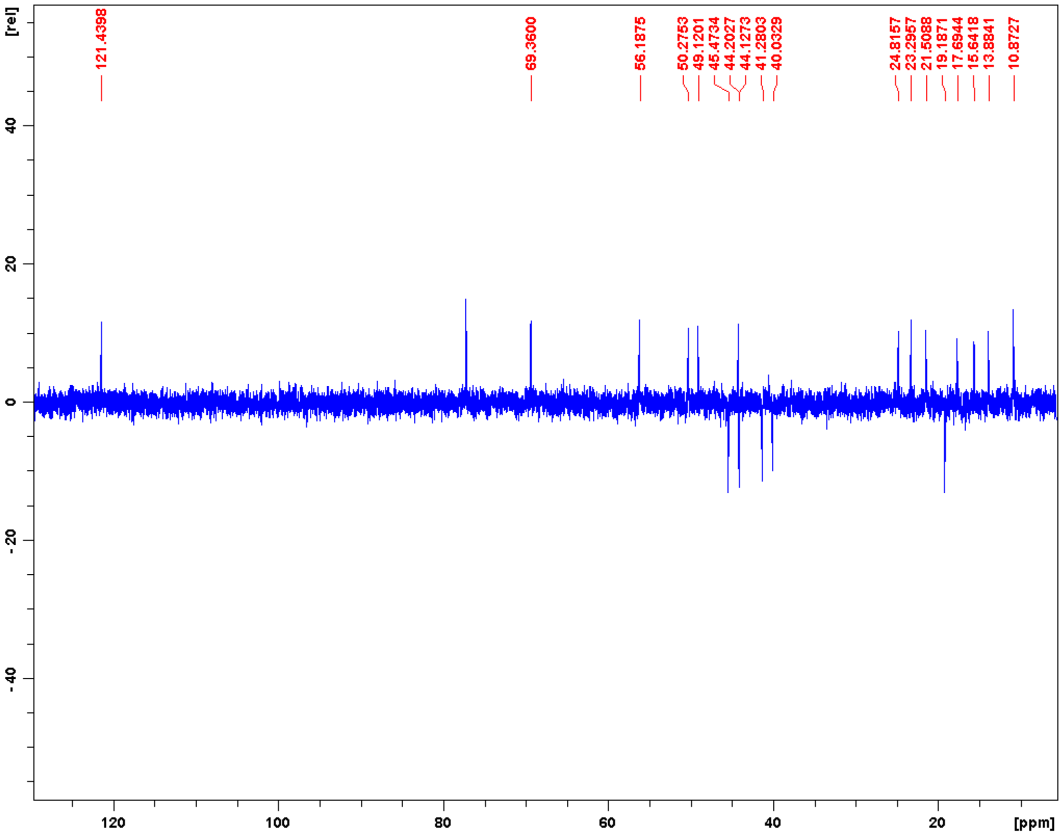

Figure S13. DEPT90 Spectrum for Compound **2**.

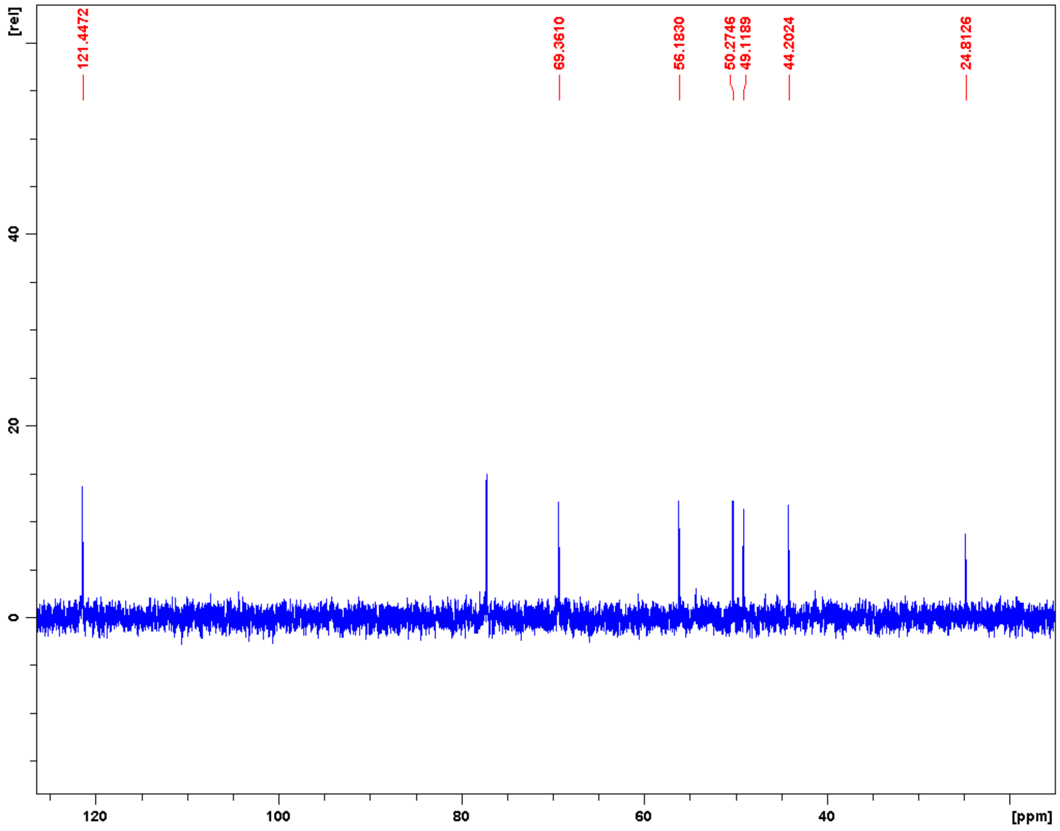

Figure S14. HMQC for Compound **2**.

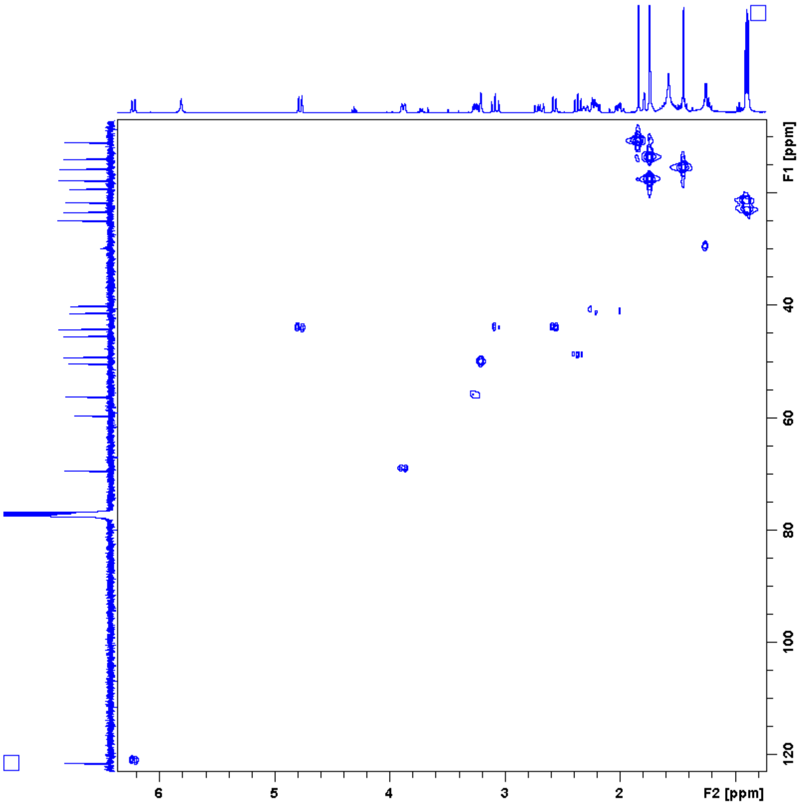

Figure S15. HMBC for Compound **2**.

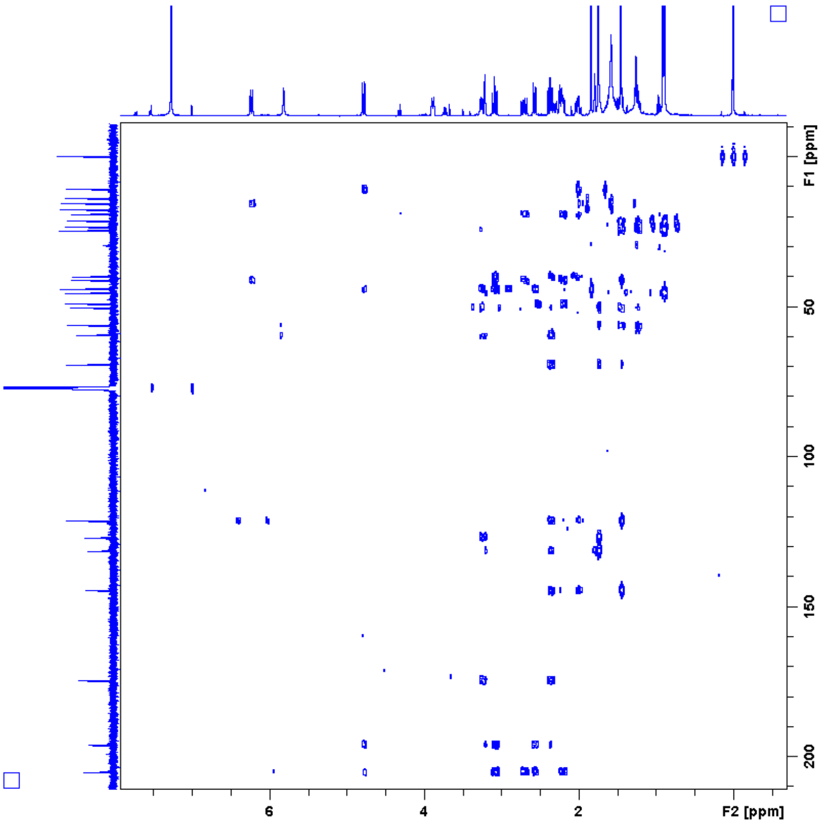

Figure S16. NOESY for Compound **2**.

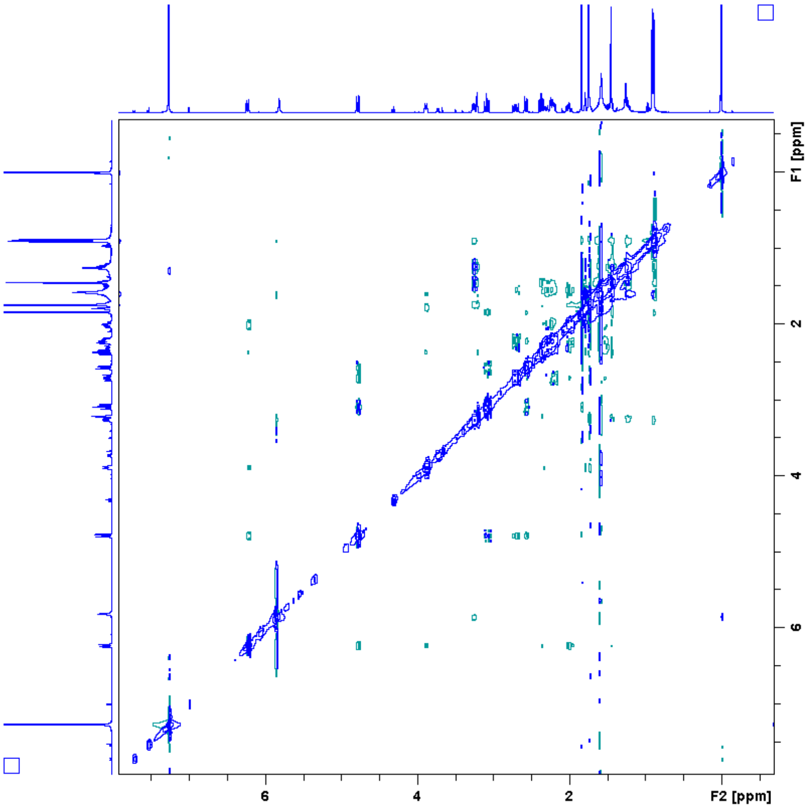

© 2014 by the authors; licensee MDPI, Basel, Switzerland. This article is an open access article distributed under the terms and conditions of the Creative Commons Attribution license (http://creativecommons.org/licenses/by/4.0/).
